# Left iliac fossa sigmoidectomy with mechanical anastomosis in the management of uncomplicated sigmoid volvulus: an observational study at Principal Hospital of Dakar, Senegal

**DOI:** 10.11604/pamj.2024.49.60.42676

**Published:** 2024-10-29

**Authors:** Eugene Gaudens Prosper Amaye Dieme, Birame Ndiaye, Magatte Faye, Samba Tiapato Faye, Moustapha Diop, Madawas Mboup, Ibrahima Sall, Oumar Fall, Alamasso Sow

**Affiliations:** 1 Department of Visceral Surgery, Principal Hospital of Dakar, Dakar, Senegal,; 2 Department of Urology, Principal Hospital of Dakar, Dakar, Senegal,; 3 Department of Infectious Diseases, Principal Hospital of Dakar, Dakar, Senegal

**Keywords:** Sigmoid volvulus, sigmoidectomy, left iliac fossa incision, mechanical anastomosis

## Abstract

**Introduction:**

sigmoidectomy is the definitive treatment of Sigmoid Volvulus (SV). It can be done either by laparotomy or laparoscopy. Our objective was to describe the left iliac fossa sigmoidectomy with mechanical anastomosis recently introduced in our practice, assess our results after 5 years and evaluate its feasibility in our setting.

**Methods:**

we conducted a prospective, descriptive and analytic study on all patients admitted for uncomplicated SV with successful non-surgical decompression and treated by a left iliac fossa sigmoidectomy with mechanical anastomosis. This study was held, from May 2016 to May 2021, at the Visceral Surgery Department of Principal Hospital of Dakar, Senegal. We studied the demographic variables, the data of the preoperative planning (time between sigmoid decompression and surgery, moment of the sigmoidectomy, mechanical bowel preparation or not, type of anesthesia), the peroperative findings (length and diameter of the sigmoid loop), the surgical procedure (the type of staplers used for the mechanical anastomosis, the duration of the operation, incidents or accidents during sigmoidectomy), the immediate and long-term postoperative course.

**Results:**

we collected 53 patients with a mean age of 50 years ± 17. They were 50 men and 3 women. Mechanical colonic preparation was performed in 18 patients (Group 1) and 35 patients did not benefit from a mechanical bowel preparation before surgery (Group 2). The mean length of the sigmoid loop was 74.5cm ± 16.5. The mean diameter of the descendant branch was 7.8cm ± 0.7 for Group 1 and 5.5cm ± 1 for Group 2 with p = 0.01. One linear cutter stapler was used for the side-to-side anastomosis. It was a 100mm in 43% (n=23) of cases. The terminalization of the side-to-side anastomosis was performed with 1 linear stapler in 37 cases, 2 linear staplers in 15 cases and 3 linear staplers in 1 case. The median duration of the operation was 50 minutes for Group 1 and 37 minutes for Group 2 with p = 0.004. Morbidity was nil in Group 1. In Group 2, we had 1 anastomotic leakage and 1 anastomotic stenosis. Mortality was nil in the 2 groups. The mean hospital stay was 5 days ± 3.7. The mean follow-up was 31 months with no recurrence or incisional hernia.

**Conclusion:**

this surgical method is rapid, simple, reproducible and feasible in our setting with a good postoperative course. Colonic mechanical preparation may not be necessary.

## Introduction

Sigmoid volvulus (SV) is the torsion of a long redundant sigmoid colon around its mesenteric axis causing colonic obstruction by strangulation [1]. This pathology is rare in Western countries; however, its incidence is high in African, Asian, South American and Middle Eastern countries where SV occurs in younger healthy patients around the 4^th^ and 5^th^ decade of life [2-6]. In these countries, SV is one of the leading causes of colonic obstruction and the main indication of colonic resection [4-6]. Sigmoidectomy is the definitive treatment of SV and prevents recurrence which can occur up to 67% if the sigmoid resection is not performed [3,7,8]. Sigmoidectomy can be performed in emergent or elective situation. Elective sigmoidectomy can be done either by classic midline laparotomy, laparoscopic approach or mini-laparotomy incision [9,10]. In 2012, Seisen proposed the left iliac fossa mini-incision to perform the elective sigmoidectomy followed by mechanical colo-colic anastomosis as a minimally invasive approach to treat SV [11]. We recently introduced this technique in our practice.

This study aimed to describe our experience on this technique, assess our results after 5 years of practice and evaluate its feasibility in our context of low income country.

## Methods

**Study design and setting:** this was a prospective, descriptive and analytical study conducted at Principal Military teaching hospital of Dakar from May 2016 to May 2021. All patients admitted at the Visceral Surgery Department for sigmoid volvulus (SV) and treated by the left iliac fossa sigmoidectomy with mechanical colo-colic anastomosis were selected. The follow-up was done at M1, M3, M6 after surgery and then every year.

### Study population

**Target population:** we collected all the patients admitted to the emergency department for SV.

**Inclusion criteria:** we selected patients who met the following 3 criteria: - Presence on admission of an uncomplicated SV after clinical examination and CT scan investigation; - Successful non-surgical decompression of the SV: spontaneous deflation or successful rectal tube decompression; - Planned left iliac fossa sigmoidectomy with colo-colic mechanical anastomosis.

**Non-inclusion criteria:** we excluded the following patients: - Complicated SV; - Uncomplicated SV with successful non-surgical decompression who benefited from a classic midline laparotomy sigmoidectomy or refused to underwent the left iliac fossa sigmoidectomy or did not benefit from it for any other reason.

**Figure 1:**
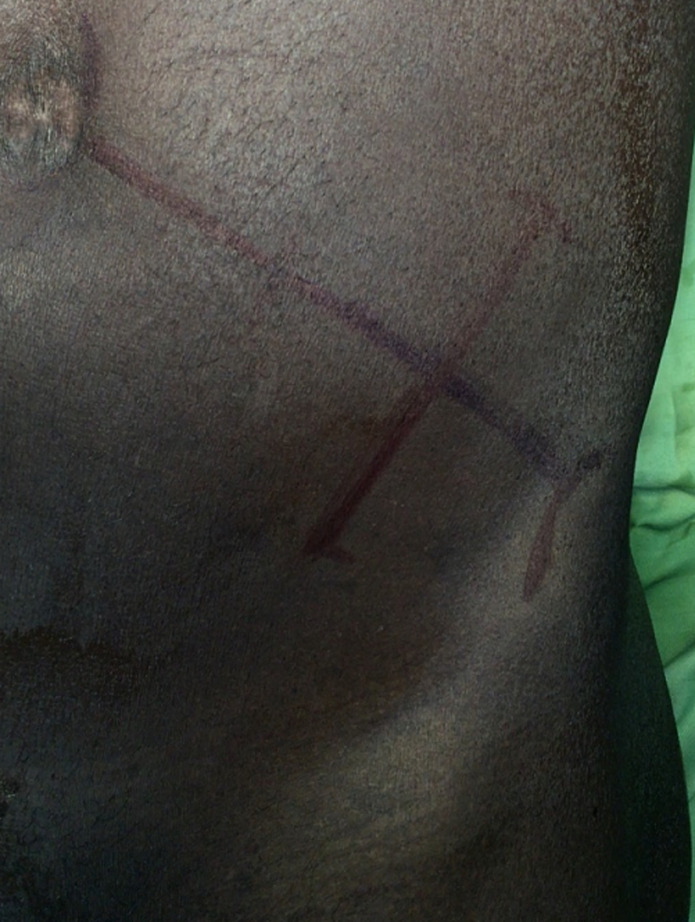
oblique skin incision at the junction of the 2/3 internal, 1/3 external in the line between the left anterosuperior iliac spine and the umbilicus

**Figure 2:**
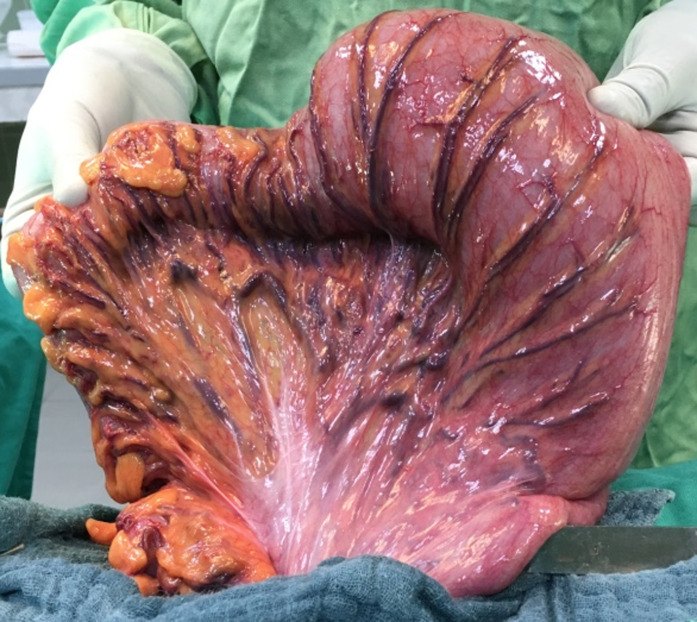
long redundant sigmoid loop entirely exteriorized by the left iliac fossa incision

**Figure 3:**
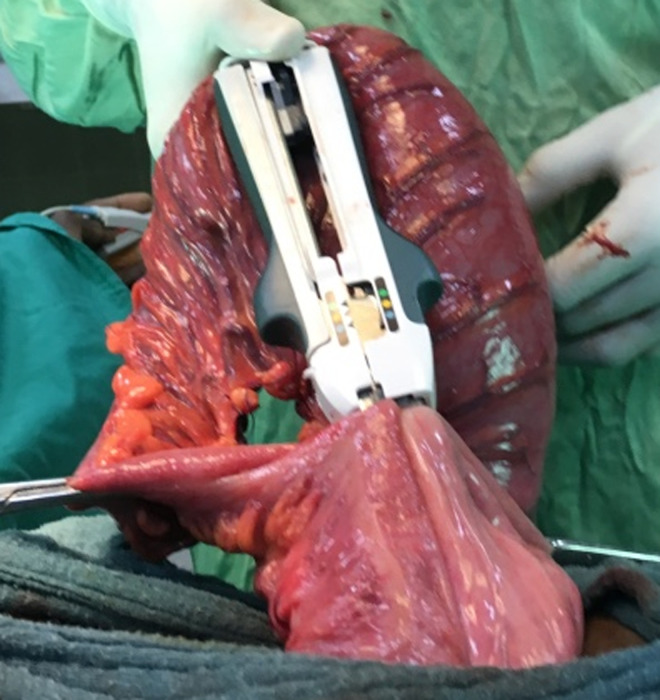
mechanical side-to-side colocolic anastomosis with a linear cutter stapler

**Figure 4:**
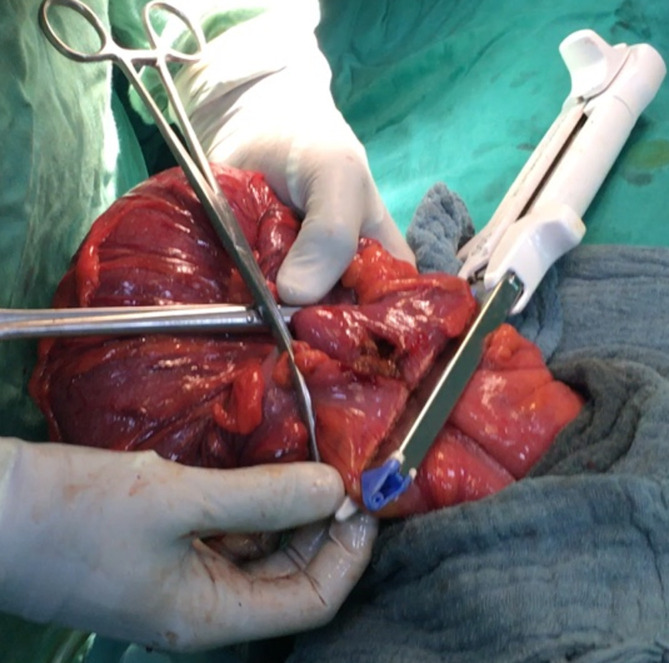
terminalization of the side-to-side colocolic anastomosis with a linear cutter stapler

**Surgical technique:** it was performed by 5 different senior surgeons. Before surgery, all the patients were on residue-free diet. A bowel preparation consisted to perform 2 bowel retrograde washout with betadine saline serum; the first the night before operation and the second the morning of the operation. Group 1 comprised the patients who had a bowel preparation and Group 2, the patients who did not have bowel preparation. The patient was installed in supine position, the surgeon at his left side, the first aid in front of the surgeon and a second aid at surgeon´s left side. General anesthesia with orotracheal intubation was performed in 21% of cases (n=11), while 70% of patients (n=37) had epidural anesthesia and 9% (n=5) spinal anesthesia. A 5 cm to 7 cm oblique skin incision was made in the left iliac fossa in the line between the left antero superior iliac spine (1/3) and the umbilicus (2/3) (Figure 1). The external oblique fascia and the underlying muscle plane were divided in the direction of their fibers followed by the incision of the peritoneum. Then the redundant sigmoid loop was externalized entirely (Figure 2). The sigmoid mesentery was divided from one border to the other of the sigmoid loop in a line above the skin surface to allow an easy colo-colic anastomosis. Two short holes were performed at the anti-mesenteric border of the 2 colonic segments to be anastomosed. The 2 anvils of a linear cutter stapler green were introduced in the colonic holes to perform the side-to-side colo-colic anastomosis (Figure 3) making sure that there is no interposition of sigmoid mesentery or epiploic fringes. The anastomosis was completed by application of a linear stapler green under the level of the 2 colonic holes of introduction of the linear stapler (Figure 4). For this, we used 1, 2 or 3 linear staplers according to the diameter of colon. A heamostatic running suture with 3-0 absorbable suture was performed along the stapler line in case of slight bleeding. The side-to-side anastomosis is reinforced with 2 or 3 sero-muscular sutures using 3-0 absorbable suture. The mesocolic defect was not closed when the terminalization of the side-to-side anastomosis was done at the level of division of the sigmoid mesentery. Then the loop was reintroduced in the peritoneum cavity. The peritoneum was closed with 3-0 absorbable suture and the external oblique fascia with slowly-absorbable sutures. The skin was closed with non-absorbable sutures or clips.

**Data collection and statistical analysis:** we studied the demographic variables (age, gender, past medical history), the data of the preoperative planning (time between non-surgical decompression and sigmoidectomy, mechanical bowel preparation or not, type of anesthesia) the findings and procedure during operation, the immediate and long term post-operative course (recovery, morbidity, mortality, hospital stay, recurrence). According to their distribution, quantitative variables were represented using either means ± standard deviations or medians and their ranges. Qualitative variables were represented using frequency and percentages. The means of independent variables were compared using either Student´s t-test or Wilcoxon Mann Whitney test according to their distribution and variances homogeneity. The proportions were compared using Pearson chi-squared test, Pearson's Chi-squared test with Yates' continuity correction or Fisher's exact test. We compared the Group 1 and the Group 2. A p-value of less than 0.05 was regarded as statistically significant. Data analysis was performed using R software (version 4.0.3).

**Ethical considerations:** all the patients give their consent to participate to the study.

## Results

**Figure 5:**
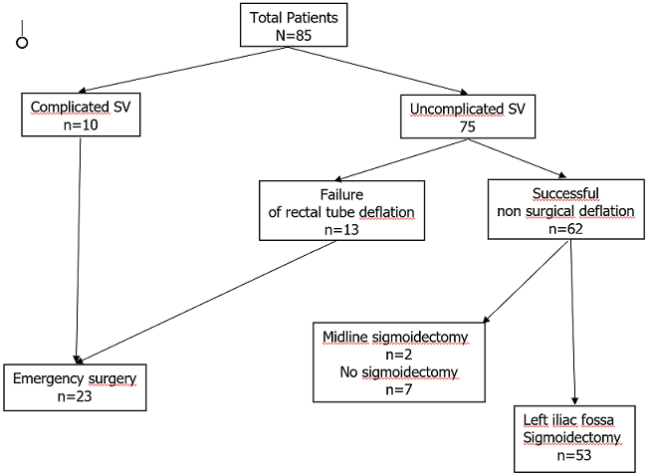
distribution of study population

**General characteristics of patients (Table 1):** from a total of 85 patients admitted, during the study period, at the emergency department for SV, we finally selected 53 patients responding to our inclusion criteria (uncomplicated SV with successful non-surgical decompression followed by left iliac fossa sigmoidectomy with mechanical anastomosis) (Figure 5). Fifty were males and 3 females. The mean age was 50 years ± 17. Chronic constipation was present in 32 patients. Three patients had high blood pressure. One had psychiatric disorder. Group 1 comprised 18 patients and Group 2 comprised 35 patients.

**Table 1:** characteristics of patients

	Mechanical colonic preparation		
	No, N = 351	Yes, N = 181	p-value2
Age	51 (19)	50 (16)	0.8
Gender			>0.9
Female	2 (5.7%)	1 (5.6%)	
Male	33 (94%)	17 (94%)	
Retractile mesosigmoiditis	62 (15)	60 (13)	0.8
Downstream diametre	5.56 (1.01)	7.83 (0.76)	0.016
Upstream diametre	4 (0)	4 (1)	0.12
Staplers for terminalisation			0.3
1 Stapler	26 (74%)	11 (61%)	
2 Staplers	9 (26%)	7 (39%)	
Duration of intervention	37 (7)	51 (18)	0.001
Hospital stay	5 (5)	5 (1)	0.6
Morbidity			0.5
No	33 (94%)	18 (100%)	
Yes	2 (5.7%)	0 (0%)	
Mortality			
No	34 (100%)	18 (100%)	
Gas transit	2 days (1)	1 day (1)	0.3

1 Mean (ET); n (%) 2 Wilcoxon-Mann-Whitney test; Fisher exact test; khi-2 test

**Surgical procedure and peroperative findings:** the median time of surgery after successful decompression was 9 days IQR 4: 42 patients (79,2%) were operated in the same admission and 11 patients (20,7%) were operated in a second admission. The mean length of the sigmoid loop was 74.5 cm ± 16.5. The mean diameter of the ascending branch of the sigmoid colon was 4 cm ± 1 for Group 1 and 4 cm ± 0 for Group 2 with p=0.12. For the descending branch, the mean diameter was 7.8 cm ± 0.7 for Group 1 and 5.5 cm ± 1.01 for Group 2 with a statistically significant difference (p=0.016). The side-to-side colo-colic anastomosis was performed with a single linear cutter stapler of 100 mm, 75 mm, 80 mm, 60 mm, 55 mm respectively in 43% (n=23), 32% (n=17), 15% (n=8), 8% (n=4) and 2% (n=1) of cases. To complete the side-to-side anastomosis, 1 linear stapler was used in 37 cases, 2 in 15 cases and 3 in 1 case. An heamostatic running suture with 3-0 absorbable suture was performed along the stapler line in 32 (60%) cases. The mean operated time was 41 minutes ± 13. For Group1 it was 51 minutes ± 17.5 and 37 minutes ± 6.7 for Group 2 with a statistically significant difference (p=0,001).

**Postoperative course and prognosis:** eight (15%) patients were allowed to take water the evening after operation, 39 (74%) day 1, 5 (9%) day 2 and 1 (2%) day 3. Light meal was introduced day 1, day 2 and day 3 respectively in 7 (13%), 41 (77%) and 5 (10%) cases. Six (11%) patients had their gas the day of surgery, 25 (47%) at day 1, 11 (21%) at day 2, 9 (17%) at day 3, 1 (2%) at day4 and 1 (2%) at day 5 after surgery. The mean length of hospital stay after sigmoidectomy was 5 days ± 3.7. The mortality was nil. We noted 1 anastomotic leakage on postoperative day 11, treated by midline re-laparotomy and closure of the fistula by interrupted 3-0 absorbable suture; and 1 anastomotic stenosis at 6 months treated by midline re-laparotomy, resection of the stenotic colon and end to end manual anastomosis with 3-0 absorbable running suture. These 2 complications occurred in Group 2. The mean follow-up was 31 months ± 17. There was no recurrence and incisional hernia. Two patients were lost during the follow-up.

## Discussion

With this prospective study, we evaluated and shared our experience on the left iliac fossa sigmoidectomy with mechanical anastomosis, a knewly introduced surgical approach to treat uncomplicated SV in our practice. We investigated 53 consecutive patients with uncomplicated SV treated by this technique in a low-income country. We did not have any problem for the availability of the staplers. The surgical procedure was efficient, safe and technically not challenging, in term of surgical approach, access to the sigmoid loop, performing the sigmoidectomy and the colo-colic anastomosis. The results were very satisfactory in term of post-operative course with a scar cosmetically appealing. The treatment of choice of uncomplicated SV is an endoscopic decompression followed by early planned elective sigmoidectomy [3]. For this elective sigmoidectomy, classic midline laparotomy has been the main surgical approach but laparoscopic approach or mini-laparotomy incision are currently used with mechanical anastomosis [9,10,12,13].

In our hospital, uncomplicated SV is treated first by instrumental detorsion using Faucher rectal tube with a success rate of 62% to 86% [5,14]. This led us to perform, during many years, sigmoidectomy in elective situation by classic midline laparotomy with hand-sewn anastomosis [5,14]. However, in sub-Saharan Africa, particularly in our sub-region, where SV is the main indication of colectomy, as endoscopic decompression is not often available in emergency, sigmoidectomy is performed in emergency regardless to the viability of the sigmoid [6,15]. This sigmoidectomy is mainly performed by classic midline laparotomy with significant mortality and morbidity, even if patients are quite young and fit [6,15,16].

From May 2016, we have adopted the left iliac fossa sigmoidectomy with mechanical anastomosis, described by Seisen in 2012, as it appears to be a minimally invasive laparotomy approach for the treatment of uncomplicated SV after non-surgical decompression [11]. To our knowledge very few series have focused on this technique, particularly none in sub-Saharan Africa and our cohort seems to be the largest [1,10]. Basato *et al*. have already compared this technique with the laparoscopic approach and have found, even though there was no difference statistically significant, higher recurrent rate and anastomotic leakage in laparoscopic group and similar thirty-day morbidity and mortality in both groups [1]. These results should be taken into account because many authors, have found some limitations with laparoscopy: difficulties of exposure due to the length and the dilation of the sigmoid, high operation time, high recurrent rate due to a poor evaluation of the level of colonic resection [1,9].

The skin incision in Seisen's technique is an oblique incision, miror to the Mac Burney incision, in left iliac fossa. In accordance with Seown and Al Dhaheri, we noted that this approach was quite easy and rapid to perform with less abdominal wall damage [1,10,11,17]. It gave us quick access to a long redundant sigmoid and allowed us to bring it out entirely. Hence, with this surgical approach, the good length of sigmoid to resect could be well assess which prevent the occurrence of recurrence. It has also a good post-operative scar comestically appealing. Others skin incision, like a short horizontal left lower quadrant incision, have also been described [13,18]. But to our concern, they seem to give more abdominal wall damage, are less rapid to perform with poorer exposure. Once the good length of sigmoid is resected, the colo-colic anastomosis could be manual [13]. However, mechanical anastomosis is quite easier to perform, it reduces the risk of fecal contamination of the surgical field, and helps, above all, to overcome the constraints of anastomosing two colonic segments of incongruent diameter [1,10]. Indeed, as shown by our study, this disparity of diameter between the 2 colonic segments was present most of the time. Like many other authors, we performed a side-to-side colo-colic mechanical anastomosis terminalized according to Seisen [10,11,17,18]. It seems to expose less to the risk of fecal contamination than the end-to-side stapled anastomosis already proposed by Chandrasekaran [19]. The terminalization of the side-to-side anastomosis is an important step in this technique. The 2 colonic segments must be well spread out for proper application of the linear stapler. As shown by our study, 1, 2 or even 3 linear staplers may be used, according to the diameters of the 2 colonic segments. In the preoperative planning, 34% of our patients had a mechanical bowel preparation (Group 1) which was associated with a higher diameter of the downstream colonic segment and a longer duration of operation time compare with those who did not have this bowel preparation. Similarly, the rate of use of 2 or more staplers for terminalization was higher in Group 1. This mechanical bowel preparation was systematic at the beginning of our study, but so far, we have abandoned it. In fact, this mechanical bowel preparation is not recommended anymore for elective colonic surgery [20]. The different series report, as us, good outcomes in term of immediate and long term post-operative course [1,10]. We think that the anastomotic leakage and stenosis that occurred in our series were related to a technical default of the terminalization. Indeed, they occurred in our first patients at the start of our use of this technique.

However, our study presented some limitations. Indeed, it was a single-center study, on a short series. We also had 5 different surgeons, with different practices. This explains partly the lack of uniformity on the size of staples used for the side-to-side colo-colic anastomosis but also on the time of water or food intake after sigmoidectomy. That´s why we planned to continue the study, extend it to other centers if possible, with well standardized procedures especially in the perioperative care of patients (no more bowel preparation, same time for introduction of water and light food after surgery) and in the surgical procedure (use of a same size stapler for the side-to-side colo-colic anastomosis for all the patients). However, the intervention of several surgeons showed that it was a very easy technique to master.

## Conclusion

This surgical technique is easy to perform, efficient, safe, reproducible and feasible in our context. It may be added in the surgical methods to treat SV in elective situation in our setting being associated with good outcome. Based on these good results, this technique is now the method of choice in our practice.

### What is known about this topic

- Sigmoid volvulus is one of the leading causes of the large bowel obstruction in Africa;
- The treatment of uncomplicated sigmoid volvulus is endoscopic decompression followed by sigmoidectomy to prevent recurrence;
- Sigmoidectomy is mainly done by classic midline laparotomy with hand sawn anastomosis in Africa.

### What this study adds

- Left iliac fossa sigmoidectomy with mechanical anastomosis is simple, safe and easily mastered;
- Left iliac fossa sigmoidectomy with mechanical anastomosis is feasible in our setting;
- Mechanical bowel preparation may not be necessary.
